# Lameness1Presented by title before the Thirty-eighth Annual Meeting of the American Veterinary Medical Association.

**Published:** 1901-10

**Authors:** W. C. Fair

**Affiliations:** Cleveland, Ohio


### LAMENESS.[1]

By W. C. Fair, V.S.,
CLEVELAND, OHIO.

Perhaps I could have selected a more interesting subject, but none more important to the veterinary profession and to the public than "Lameness in the Horse." I believe that it is not receiving the careful study and consideration that some other branches of our work are receiving. One-fourth of my practice during the past thirty years has been with lame horses, and I estimate that one-tenth of the horses in the city of Cleveland are either lame or sore, and I apprehend that this proportion will be found true in all other cities of the country; hence the necessity of more profound study by veterinarians. I may not be able to enlighten many of you, but I myself may gain some valuable information by its discussion.

What is lameness? Lameness is a manifestation of pain by one or more limbs, weakness, inability, always indicating soreness, stiffness, causing partial or total inability to use the limb or limbs. Now, in order to detect lameness you must be conversant with the natural gaits and all their peculiarities. Without this knowledge no one can tell the difference between lameness and peculiarity of action. Many young horses appear lame when being led by a short rein and head pulled to one side; a horse that is habitually led or exercised in a ring or round a circle always appears lame in the limb nearest the centre of the circle; many horses travel with a peculiar nod to their head, and hitch or hop behind—all of which baffles the inexperienced person. It is not always easy to deter-

[1] Presented by title before the Thirty-eighth Annual Meeting of the American Veterinary Medical Association.

mine whether the lameness is in a fore- or a hindlimb, for example: If a horse lame in the off forelimb is trotted from you he seems to be lame in the near hindlimb, for that quarter ascends and descends. Trot the animal toward you, and it will be seen that the irregular motion of the hindquarters is due to the elevation and depression of head and body and that the lameness is in the forward limb. Trot the horse both ways—to and from you—before deciding. Another difficulty occurs when the trouble is in both forefeet. In such a case the animal may go as though sound; he may not nod his head, but will be short in action. When lameness is in both hindlimbs the animal is both *stiff* and *lame*, but mere stiffness should not be confounded with lameness, as it frequently is.

Lameness may arise from disease apart from the limbs, as from injury to or disease of the spinal cord or nerves or liver; it may also arise independently of disease, as in cases of stone-bruise; the animal evinces pain, while there is no disease going on within the foot. It is also the case many times after a shoeing, when the shoe has been poorly fitted. If these causes exist for any great length of time inflammation is sure to follow. There are some forms of lameness apparent in the stable only, hence the necessity of seeing a horse led out, as many forms of lameness disappear after a few steps are taken. In order to ascertain correctly whether a horse be lame, approach him quietly and when he is free from all excitement.

The signs of lameness during repose are very important and frequently assist materially in diagnosing a case. If resting on all four limbs, the position of the lame one will usually be more upright than the others. Should one foot be a few inches in advance of the other it indicates soreness in the heel or back part of the foot, and is done to relieve the affected part. Bending of the knees and fetlock and resting the foot on the toe, without any advance of the other, usually implies trouble in the shoulder or elbow. If both forefeet are kept in advance of the body we have a right to suspect foot-lameness and soreness. In many cases of foot-lameness he will show it most when allowed to stand still. If both forefeet are involved, he will certainly change from one foot to the other. One horse may leave the stable very lame and recover from it entirely after a little exercise, while another may come out sound and become lame from the exercise. Lameness that shows itself when a horse is turned around is very hard to detect; some will show it only when sharply turned. Slight cases of stringhalt or chorea are seldom detected except during the turn; some show lameness in turning one way, and some the other, and others walk down hill as though lame, even though sound. The pointing in elbow-lameness is characteristic, the forearm being extended, the knee in a state of flexion. In severe shoulder-lameness the animal usually points backward—the limb is relaxed, the knee bent, and the foot placed back of the opposite one. Sometimes the toe scarcely touches the ground. If lameness be in the hind limb the horse may stand with the limb flexed or knuckling over the fetlock, or with the foot lifted clear off the ground. If the horse stands with the lame hind leg in advance of the sound one it generally indicates disease in or below the hock. Disease much more frequently exists in a limb without lameness than with lameness without disease; in fact, lameness is seldom present without pain. However, in case of a dislocated patella he is too lame even to move, or in cases of complete ankylosis; in neither case does he suffer pain, but is very lame.

In the detection of lameness you will observe, when the limb comes to the ground during progression, the animal suddenly elevates that side of the body and drops the other side. If the lameness be in a forelimb the head as well as the forepart of the trunk is raised from the lame limb and dropped upon the sound one. If the lameness be in a hindlimb the quarter of the side will be elevated, and that of the sound side thrown forward and downward by a jerking motion. The head will be carried moderately steady if the pain be not great, and jerked if there be acute pain. The slow trot is the best gait by which to locate lameness. If a horse walks lame he must be very lame when trotted, and he should be trotted immediately when first taken out, as many slight lamenesses disappear after a short walk.

There are some forms of lameness which are manifested only after sharp work or a hard drive and the animal allowed to cool. When taken out of the stable and trotted, after such a rest, lameness if in existence will most assuredly be detectable. The gait only is sufficient to determine the seat of lameness, and in some cases it is the only guide. However, it is well to examine the limb carefully and to discover lameness by both positive and negative signs. For example, if there be heat, pain, or swelling in any part of the limb discoverable by manipulation, the evidence is positive that the cause is in such part; but if, on the other hand, there be neither heat, pain, nor swelling of the limb, then we must conclude that the trouble is deep-seated and in a part of the quarter thickly clothed by healthy tissues, and we must arrive at a conclusion by negative evidence, but do not be misled by peculiarities of gait.

Lameness may be caused by the strain of a muscle, tendon, or ligament, by fractures, diseased bones, cartilage, or fibrocartilage, morbid conditions of the skin, wounds, tumors, plugging of arteries, accident, pricks in shoeing, ulcers, bruises, rheumatism, and reflex nervous action.

When a muscle is strained the injury is succeeded by pain, heat, swelling, and loss of function. An inflamed muscle can no longer contract, hence in strains the symptoms resemble those of paralysis. The swelling of an inflamed muscle is often succeeded by atrophy and loss of function, and its power completely destroyed, and if the whole muscle be involved its contractile power no longer exists. Perhaps the worst cases of atrophy follow azoturia, where we have, first, an inflammation of muscles, followed by atrophy; recovery in all such cases is slow. However, atrophy of the shoulder is very common. Counter-irritation, massage, and generous feeding, with walking exercise (if the animal be not lame) will prove beneficial.

A sprain of a ligament or tendon should be regarded as a serious injury. A tendon being of low vitality recovery is always slow and uncertain, and it rarely ever regains fully its normal condition. Perhaps this is due to the early treatment being wrong. How are strained ligaments and tendons generally treated? Certainly, the animal is not given absolute rest, and that is the important thing to do. It is true, good results follow the use of both hot and cold applications, plaster casts, and slings; also mild as well as severe counter-irritants, but *rest* is the most essential. When a lame animal is exercised his pain usually increases. Therefore, it is humane to give him rest. Weak and lame horses need no exercise. All sprains require rest, and if the cause of lameness can be ascertained then its removal will be a stride in the right direction in treatment.

An error which is frequently committed is the turning out of a lame horse to pasture. He is obliged to exercise as he picks his living. Another mistake is the exercising of trotting and running horses on race-tracks, thus preventing them from making satisfactory progress toward recovery. By exercising a lame horse we retard recovery and frequently produce atrophy of the healthy muscles in the same limb. If hard pulling or fast driving causes lameness, why not remove the cause and give him absolute rest? That is the very reason why so many track-horses never fully recover. They are not allowed to rest after meeting with an injury. True, exercise will develop muscles, tendons, and ligaments, provided the animal be not lame and weak in any one quarter.

In order to be able to treat lameness successfully we should be familiar with all causes as well as symptoms and signs. By being thoroughly conversant with these we know where to look for lameness. One of the most common causes of lameness is improper shoeing or leaving shoes on too long. How many horses have too much of the soles of their feet removed when shod? How many of them are shod level? How many of them are obliged to go on calks? How many suffer from corns, quarter-cracks, navicular disease, sidebone, ringbone, splints, and sore tendons—the result of high heels?

It is a fact that 50 per cent. of our city horses suffer from navicular joint trouble after three years travel on our paved streets. Can we not prevent this by prevailing upon the horseshoer to leave the sole of the foot alone—allow it to remain strong instead of weakening it, causing it to become bruised? Also insist upon his shoeing the horse level instead of raising his heels much higher than his toe; also, to apply protection to the sole and heels in the shape of rubber pads, which very often prevent puncture, relieve pain, and reduce lameness. Rubber pads and rubber-and-metal shoes should be used to prevent soreness and disease as well as to relieve it. I am also satisfied that if our city horses were better shod their usefulness would be lengthened very materially, and, much as I dislike to say it, poor horseshoeing is responsible for a great deal of lameness. Now, what are the results of shoeing a horse *not level*? It will have a tendency to cause soreness, sidebone, splint, ringbone, spavin, corns, and navicular joint trouble. It is true that navicular lameness occasionally follows long periods of rest, inactivity, and irregular exercise, perhaps due to a deficiency of synovial secretion and no moisture being supplied to the foot. Very few horses suffer from navicular disease where the heel is kept low and the frog allowed to come in contact with the ground at each step. Raising the heel is altogether too common a practice in shoeing, and I have observed that it is the cause of much foot-lameness. The first indication of navicular trouble is a peculiar shifting of the feet and a shortness of the step, increased heat about the heel and coronet, a disposition to point, a dryness of the hoof, some throbbing of the plantar arteries, and pain on pressure in the hollow of the pastern; also, a general tenderness of the foot on pressure, especially the back and lower part under the navicular joint. Contraction of the foot always succeeds navicular disease, and by removing too much of the sole of the foot and bars and frog it induces contraction and is a fruitful source of this complaint. The atrophy following this disease is not always confined to the foot, as we frequently find atrophy of the muscles of the shoulder and forearm.

Another very common cause of lameness is laminitis or inflammation of the feet. True, it is not always confined to the feet, but its chief seat is there. Weak-footed horses that are used on pavements and hard roads where they are driven fast frequently suffer from a mild form of laminitis, and when it does occur in a chronic form it produces a soreness and lameness that are troublesome to relieve. This condition is frequently the result of thinning the sole, thereby allowing the bottom of the foot to become bruised. The remedy in such cases is to protect the foot, prevent concussion, and apply moisture, and it is well to observe the condition of his digestive organs. An animal suffering from chronic indigestion usually has brittle and somewhat unhealthy hoofs.

Another very common cause of lameness is striking the shin with the opposite foot. About 25 per cent. of all shod horses do interfere more or less. This statement is not at all flattering to the average horseshoer, but it is a fact, nevertheless, and it is certainly one of the common causes of lameness. The remedy is a change of shoer or protect the shin and fetlock with boots.

We also find a great deal of lameness in the hock-joint, caused by improper shoeing and driving young animals on hard footing, but no doubt many of them inherit a predisposition to disease of the hock-joint. Lameness in this joint is usually easily detected by peculiarity of action, and the treatment of hock-lameness is generally satisfactory in young animals, but not in old ones.

Lameness being an evidence of pain, is it not the duty not only of the veterinarian but of the different humane societies to insist upon lame animals being rested? By resting them their chances of recovery are greatly enhanced, besides, their owners will perhaps lose less in the end. It is astonishing how anxious owners are to use lame horses, especially on the turf. Owners of such animals are usually unreasonable, and do not deem it necessary to rest a lame horse.

In this paper I have briefly pointed out the signs of lameness and peculiarities of gait and a few of the most common causes, and, in conclusion, let me give the young practitioner a word of advice.

When you are called to see a case of lameness, or when a lame horse is brought to you for examination, do not give an opinion too hastily. Try to persuade the owner to leave the horse at your hospital; examine him critically; be sure you are right before you give an opinion; at the same time try to ascertain the cause and remove it. Very few veterinarians are so expert in the diagnosis of lameness as to be able to give a correct opinion without making a close examination. However, there are exceptions to this rule.

Regarding the examination of horses as to soundness: Never give an opinion until you have examined the horse after he has stood a considerable time. There are many slight lamenesses that disappear with exercise. Turn him both ways, watch him closely on the turn; you may be able to detect lameness. If you make a mistake someone must know it, and it will certainly injure your reputation. What are the common practices of veterinarians? They look at the animal casually and give an opinion without being positive that the animal is not lame or sore. Many practitioners are exceedingly careless in the examination of lame horses. My clients are frequently displeased at me because I will not give them an off-hand opinion after a very hasty examination. However, by refusing I do not lose the case, but am allowed time to make a careful examination. By so doing I protect my reputation. After spending thirty years of my life in active practice, with perhaps 25 per cent. of my business lameness, I do not give as hasty an opinion as I formerly did, knowing full well how easy it is to make a mistake. You may be able to tell at a glance whether a horse is lame in the shoulder or in the foot; but what causes the lameness is another proposition. Examine closely each case that comes to you, and insist upon having sufficient time to make a thorough investigation before giving an opinion.

